# Fatty Acid-Binding Proteins in Psoriasis—A Review

**DOI:** 10.3390/metabo12090833

**Published:** 2022-09-03

**Authors:** Julia Nowowiejska, Anna Baran, Iwona Flisiak

**Affiliations:** Department of Dermatology and Venereology, Medical University of Bialystok, Zurawia 14 St., 15-540 Bialystok, Poland

**Keywords:** fatty acid-binding protein, FABP, psoriasis, metabolic syndrome, liver fatty-acid-binding protein, muscle heart fatty-acid-binding protein, adipocyte fatty-acid-binding protein, epidermal fatty-acid-binding protein, psoriasis-associated fatty-acid-binding protein, brain fatty-acid-binding protein

## Abstract

Psoriasis is one of the most common skin diseases in dermatological practice. It affects about 1–3% of the general population and is associated with different comorbidities, especially metabolic syndrome. Fatty-acid-binding proteins (FABPs) are a family of cytosolic proteins which are an important link in lipid metabolism and transport; moreover, they have different tissue specificity and properties. So far, ten FABPs have been discovered and seven have been investigated in psoriasis. In this review, we discuss the nature of all FABPs and their role in psoriasis. FABPs have different organ and tissue expression, and hence various functions, and may be markers of different disorders. Considering the concentration of a few of them tends to be elevated in psoriasis, it confirms the current perception of psoriasis as a multiorgan disorder associated with plenty of comorbidities. Some FABPs may be also further investigated as biomarkers of psoriasis organ complications. FABP-1 and FABP-5 may become potential markers of metabolic complications and inflammation in psoriasis. FABP-7 could perhaps be further investigated as an indicator of the neurodegenerative processes in psoriatic patients.

## 1. Introduction

Psoriasis is one of the most common skin diseases in dermatological practice. It affects about 1–3% of the general population and even up to 125 million people worldwide [1]. There are many clinical types of psoriasis, of which the most common is plaque psoriasis [2]. It manifests as erythematous-infiltrative plaques with a scaly surface, which may be localized anywhere on the body, but the most frequently affected areas are extensor surfaces of the knees, elbows, lumbosacral area, or scalp [2]. Lesions can be accompanied by pruritus, burning sensations, or even pain [2].

The pathogenesis of psoriasis still remains uncertain. The contemporary state of knowledge suggests that it is genetically determined, but additionally, immunological disturbances, including autoimmune, occur [2]. Moreover, environmental factors may trigger the onset of psoriasis or exacerbate the skin lesions’ severity [2]. From the histopathological point of view, psoriasis is characterized by the hyperproliferation of epidermal cells, parakeratosis, and neutrophil infiltrations in the epidermis [2,3]. Because of the unesthetic appearance of the lesions, psoriasis has become a source of great psychological burden for the patients and a real problem in their psychosocial life [2].

So far, there has been no successful treatment for psoriasis that would lead to healing of the lesions definitively and irreversibly; hence, psoriasis remains an incurable disease and a great therapeutic challenge [4]. Nowadays, available therapies include topical treatment (e.g., glucocorticoids and calcineurin inhibitors), phototherapy, classic systemic treatment (methotrexate, acitretin, cyclosporin A, apremilast, and dimethyl fumarate), and biological treatment (antibodies targeting cytokines or their receptors engaged in psoriasis pathogenesis) [5]. Unfortunately, access to the latter is still limited in many countries.

Although psoriasis has been considered just a skin disease for a long time, nowadays, we know that it is also associated with many other disorders which shorten patients’ life duration and worsen their quality of life [6,7]. This is why psoriasis is so widely investigated in relation to its different comorbidities, their markers, and possible detection of complications.

Psoriasis has been linked to many other conditions, such as arthritis, inflammatory bowel diseases, kidney and lung diseases, or psychological disorders, but one of the most important comorbidities of this dermatosis remains metabolic syndrome (MS) [8]. Psoriatics have been proved to suffer more frequently from obesity, arterial hypertension, diabetes mellitus, and dyslipidemia [9]. Noteworthily, all of these associations are bidirectional, meaning psoriasis predisposes to metabolic disorders and they may also predispose to or exacerbate psoriasis [9,10]. Interestingly, drugs that are used in the therapy of metabolic disorders are also known to be successful in the improvement in skin condition in psoriatic patients. Such observations have been made, for instance, for statins and fibrates [11,12]. Therefore, the search for metabolic disorders’ markers application in psoriasis has been widely studied. Multiple pieces of research have proved the possible use of different cardiometabolic complications’ biomarkers also in psoriasis [13,14]. One such group of biomarkers is serum concentrations of particular fatty-acid-binding proteins (FABPs). However, the role of some of them can go beyond MS.

## 2. Fatty-Acid-Binding Proteins

**Figure 1:**
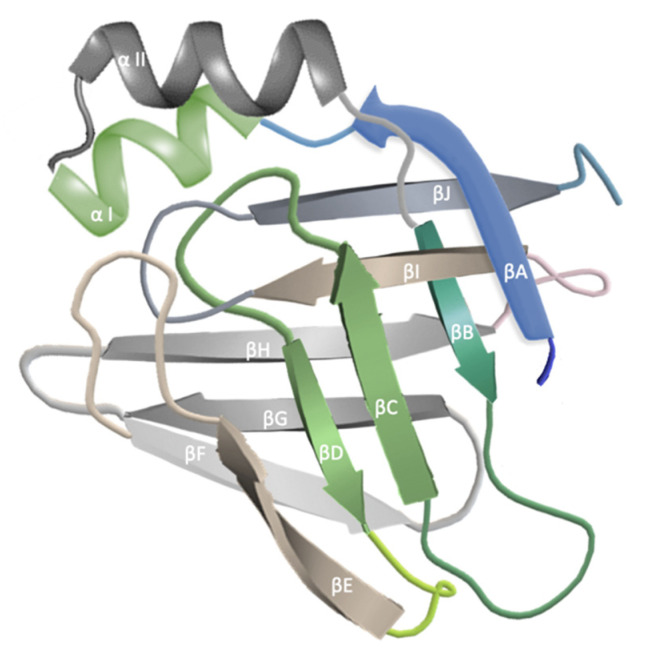
General overview on FABPs’ structure, based on [17,18,19].

FABPs are cytosolic proteins that are an important link in lipid metabolism and transport [15]. They have been named after the tissue that they were found in and also given consecutive numbers [15]. So far, there have been ten FABPs described [16]. They vary in the distribution in particular organs and, hence, their function. All FABPs have the capability of binding hydrophobic lipid ligands in the cavity of the β barrel structure, which is composed of 10 antiparallel β stands and capped by a helix–turn–helix motif [15] (Figure 1). The differences between each FABPs and, hence, their ligand-binding specificity and affinity are determined by their amino acid sequences [15]. 

They exert a pleiotropic function since they take part in the solubilization, transport, and metabolism of fatty acids; they have interactions with different membrane and intracellular proteins and adjust various lipid responses [15]. FABPs protect cells from the damaging influence of the accumulation of long-chain fatty acids [15,20].

FABPs have been already investigated in psoriatic patients, mainly due to their association with metabolic disorders, but also, proteins that are not related to such disturbances have brought some interesting and relevant results. Nevertheless, there have been no reports so far that would discuss all FABPs together in relation to psoriasis. Hence, we would like to perform, for the first time, a thorough review of their role in this dermatosis to present what is already known about the topic and how FABPs may be meaningful for psoriasis management, as well as point out knowledge gaps and potential future research directions.

## 3. Fatty-Acid-Binding Proteins in Psoriasis

FABPs have already gained attention in psoriatic patients. Out of ten described proteins, seven have been investigated in psoriasis so far. The majority turned out to be elevated in patients’ sera and some of them have been proved to be correlated with psoriasis severity or associated with treatment. Only a few FABPs have also been studied in psoriatic lesions.

### 3.1. FABP-1

FABP-1, called liver FABP [15], was the first described protein [21], and is encoded by the gene on chromosome 2p11 [16]. It is involved in the lipid synthesis, binding, and transport of long-chain fatty acids and exerts antioxidant properties [15,16,22]. It appears mainly in hepatocytes but it may also be detected in intestines and kidneys [22]. Under normal circumstances, FABP-1 is undetectable in human serum [23]. FABP-1 has been suggested as a marker of kidney injury, since its concentration increases in such conditions of different etiology [22] and lung injury, and is a predictive factor for nonalcoholic fatty liver disease (NAFLD) [21]. Its increased concentrations have been also observed in MS, including obesity and subjects with impaired glucose metabolism [23,24]. The antihyperlipidemic therapy with statins or fibrates leads to upregulation of FABP-1 expression in the liver [16].

Our team has been the only one who has investigated FABP-1 in psoriatic patients so far and its serum concentration was significantly elevated compared to subjects without skin diseases [21]. However, its serum level was not correlated with psoriasis severity expressed by PASI (psoriasis severity and activity index). FABP-1 concentration was significantly higher in obese patients compared to controls without dermatoses and overweight or normal-weight patients [21]. Moreover, FABP-1 was positively correlated with C-reactive protein (CRP) concentration [21]. After treatment with methotrexate or acitretin, FABP-1 concentration significantly decreased compared to the status before the therapy [21]. Based on this study, it may be suspected that FABP-1 could be considered a marker of metabolic complications in psoriasis and help in indicating patients who could be more prone to such disorders, which could be followed by the earlier introduction of specialized examinations and treatment in this group. Moreover, considering the current perception of psoriasis as a kind of systemic inflammatory condition [25], FABP-1 could become an indicator of systemic inflammation in such patients. Furthermore, it could be used as a predictive factor of the clinical response to antipsoriatic therapy [21]. New studies investigating its role would be helpful to expand the current state of knowledge.

### 3.2. FABP-2

FABP-2 is called intestinal FABP [15]. Its chromosomal location is 4q28–q31 [16]. FABP-2 is present in the small and large intestine and is involved in the absorption of dietary fats [15,26]. FABP-2 has been considered a marker of enterocyte damage since it is present only in these cells [27]. It was reported to be increased in serum in the case of ulcerative colitis and necrotizing enterocolitis [22]. Moreover, it has been suggested as a biomarker of abdominal, and particularly intestinal, injury in trauma patients [28]. An interesting, but still to be fully explained, observation is that FABP-2 concentrations are decreased in the sera of patients with COVID-19, independently of gastrointestinal involvement [29].

FABP-2 has already been investigated in psoriatic patients and its serum concentration was found to be significantly elevated compared to the control group, which supported the concept of the disturbed intestinal barrier in psoriatics [30]. Moreover, FABP-2 concentration positively correlated with BMI and PASI, and it was recognized as an independent predictor of psoriasis severity in patients with moderate to severe disease [27]. The authors suggested that the integrity of the intestinal barrier in patients with psoriasis plays an essential role in the pathogenesis of this dermatosis and its function may be influenced by body mass and the severity of skin lesions [27]. Therefore, FABP-2 has emerged as a possible marker of psoriasis severity and its potential use should be further elucidated. Moreover, it could be of value to examine the influence of antipsoriatic treatment (followed by a decrease in psoriasis severity) and body mass loss in relation to FABP-2 and intestinal-barrier integrity.

### 3.3. FABP-3

FABP-3 is called muscle-heart FABP [15] and its gene is located on chromosome 1p33–p31 [20]. The expression of FABP-3 is observed in the heart, skeletal muscle, brain, mammary glands, and brown adipose tissue [20]. FABP-3 is mainly engaged in the uptake and oxidation of muscle lipids [15]. Moreover, it influences glucose homeostasis [20] and thermoregulation [20,31]. FABP-3 has been once suggested as an acute myocardial infarction [16,20,32] and heart failure biomarker [33] since it can be released into the bloodstream due to cardiomyocyte injury [33].

FABP-3 has already been investigated in psoriatic patients and its serum concentration did not significantly differ from the subjects without dermatoses in two independent studies [20,34]. In the first one, its concentration did not correlate with PASI or BMI either [20]. Despite such outcomes, the same study presented a positive correlation between FABP-3 and white blood cell count and liver enzyme activity and suggested FABP-3 as an indicator of inflammation or liver pathology [20].

As psoriasis is known to be tightly related to cardiometabolic complications, the search for the use of their biomarkers in this group of patients is justified. Considering there was no significant difference in FABP-3 concentration between psoriatics and subjects without skin diseases, it is advisable to perform further studies in order to establish their definite association. Particularly, the role of FABP-3 as the marker of inflammatory processes or liver disorders in psoriasis seems promising.

### 3.4. FABP-4

FABP-4 is called adipocyte FABP [15] with the encoding gene located on chromosome 8q21 [16]. FABP-4 is involved in the storage of lipids, their metabolism, and lipolysis [15]. It is abundantly present in adipocytes, hence the name, but may be also found in endothelium or macrophages [20]. This FABP has been associated with several components of MS and is even considered a predictive marker of this disorder [20,35]. It has been suggested that increased serum concentrations of FABP-4 may occur in patients with diabetes mellitus type 2, obesity, arterial hypertension, and nonalcoholic fatty liver disease or atherosclerosis [20,22]. FABP-4 concentration has been reported to be independently associated with an alteration in carotid intima–media thickness, a marker of atherosclerosis, per one year; hence, it suggests that FABP-4 concentration could become a marker of atherosclerosis progression [36]. Of note, it has also been postulated as a predictor of long-time cardiovascular complications and mortality [36]. Moreover, based on bariatric surgery studies, FABP-4 has been proposed as a marker of dynamic body weight changes [22].

FABP-4 has already been investigated in psoriatic patients and its serum concentration turned out to be elevated compared to controls in three studies [20,34,37], especially in overweight subjects [20]. However, no direct correlation between FABP-4 concentration and BMI has been established [20,38]. There was also no correlation between FABP-4 concentration and PASI found, although its level was significantly higher in patients with mild psoriasis (PASI < 10) [20]. Based on previous studies regarding the role of FABP-4, it has been proposed as a marker of metabolic complication in psoriatics [20]. Moreover, in one of the most recent studies performed on patients with psoriatic arthritis, it was shown that FABP-4 was elevated in patients with cardiovascular diseases and correlated with higher disease activity, as well as that *FABP-4* gene expression was upregulated [39]. In another study on psoriatics, the association of FABP-4 with systemic antipsoriatic agents was investigated. After 12 weeks of systemic treatment with methotrexate or acitretin, the FABP-4 serum concentration, which was elevated in patients before the therapy, decreased, although insignificantly. However, after analysis of both administered drugs, it turned out that it was acitretin that caused a significant drop in FABP-4 concentration. Hence, the authors suggested that FABP-4 might be a predictor of clinical response to acitretin [37]. Circulating FABP-4 has also been proven to be independently associated with the concentration of PCSK9 [36]. Our team has studied this molecule in psoriatics in the past and proved that it is elevated in such patients [40], which seems consistent and points out again the relationship between psoriasis and MS.

On the contrary to the above-mentioned outcomes, in one study by Sigurdardottir et al., no statistically significant difference in FABP-4 concentration was observed between the psoriatic patients and controls [34]. In the same study, despite no primary difference before the therapy, FABP-4 concentration significantly decreased after treating the patients with narrow-band UVB [34]. There is also one more study by Honma et al. [41], described in a letter to the editor, where the scientists assessed FABP-4 in psoriatics; however, they did not include a control group for comparison. At the same time, they did not notice a correlation between FABP-4 and PASI, similar to the study by Baran et al. [37].

Considering these slightly inconsistent results, it may be beneficial to investigate serum FABP-4 on larger cohorts of psoriatics compared to the matched control group of patients without dermatoses, especially in relation to therapy, for both phototherapy and systemic agents.

### 3.5. FABP-5

FABP-5 is called epidermal or psoriasis-associated FABP [15]. The *FABP-5* gene is located on chromosome 8q21.13 [16]. Its expression is most prominent in the epidermis but also in the brain, kidneys, liver, lungs, testes, and adipose tissue [20]. FABP-5 is considered a transporting and binding agent [15], engaged in glucose and lipid homeostasis [16]. It has been also reported that it influences the differentiation of keratinocytes [20,42].

FABP-5 was identified for the first time in the psoriatic epidermis, hence the name [16]. It has already been investigated in psoriatic patients, and not only in their sera, but also in psoriatic plaques [20,43]. The serum concentration of FABP-5 has been evaluated with different outcomes. In one study by Miyake et al., it was similar in psoriatic patients and subjects without dermatoses [43]. In another study by Kozlowska et al., it turned out to be elevated in psoriatics compared to controls and positively correlated with skin lesions’ severity in PASI [44].

As for the assessment of this FABP in psoriatic skin, it has been found to be overexpressed compared to the unlesional skin in three studies [42,43,45]. Of note, its concentration was not associated with PASI in the study by Miyake et al. but it was indeed with erythema, infiltration, and exfoliation [43].

FABP-5 has been suggested as a marker of metabolic complications and, as psoriasis is tightly associated with MS, it could be proposed as such a marker in this group of patients [44]. FABP-5 has been also proven to be positively correlated with inflammatory parameters in one study on psoriatics, namely C-reactive protein and white blood cell count [44], which may indicate its use as an indicator of inflammation in such patients. However, considering some inconsistencies between the results of the only two available studies, new research is needed to further investigate this matter.

### 3.6. FABP-6

FABP-6 is called ileal FABP [15] and is hardly studied in the literature compared to other FABPs. Its chromosomal location is 5q23–q35 and predominant expression is observed in the ileum, as well as ovaries or placenta [16]. FABP-6 is involved in intestinal biliary homeostasis [15] via the intracellular transport of bile acids in the ileal epithelium [46]. FABP-6 has been proven to be overexpressed in colorectal cancer and renal cell carcinoma tissue [46,47].

To the best of our knowledge, FABP-6 has never been evaluated in psoriatic patients, so its role is yet to be discovered. However, considering biliary metabolism disorders in psoriatic patients, including less conjugated primary bile acids in their bile, and hence increased concentration of the precursors of cholesterol in plasma [48], it is probable that aberrations of this protein’s concentrations may occur in this group.

### 3.7. FABP-7

FABP-7 is called brain FABP [15] and is encoded by 6q22–q23 [16]. FABP-7 is present in the brain and has been proven to be involved in brain development and neurogenesis [15]. It is involved in the storage and transport of docosahexaenoic acid (DHA) and eicosapentaenoic acid (EPA) and also protects DHA from unfavorable peroxidation [49,50,51]. As for the pathological conditions, FABP-7 has been investigated in relation to neurodegeneration. Research has shown that it is elevated in the serum of patients with Alzheimer’s and Parkinson’s disease [22,50]. Moreover, FABP-7 has been proven to be increased in elderly patients with postoperative cognitive dysfunction who underwent spinal surgery compared to patients with no such dysfunction and before the procedure [52]. FABP-7 has been also investigated in relation to psychiatric diseases and it appeared that three single-nucleotide polymorphisms (SNPs) from FABP-7 showed nominal association with bipolar disorder [53]. The same team of scientists also proved altered expression of FABP-7 in schizophrenic brains and genetic association with schizophrenia [54]. FABP-7 has also been found to be engaged in the pathogenesis of several neoplasms, such as malignant glioma [55] or renal cell carcinoma [56] and sleep process [57].

As for the role of FABP-7 in psoriasis, our team has already investigated this matter and its serum concentration turned out to be elevated compared to controls without dermatoses [58]. We also analyzed the concentration of FABP-7 before and after the treatment with systemic antipsoriatic agents (methotrexate and acitretin) and noticed a drop, although insignificant, in its concentration after therapy, and there was no difference regarding the use of a particular agent [58]. We are not aware of other studies investigating this FABP in psoriatic patients. Results of our investigation, in combination with other obtained data, may suggest that psoriatics may be at increased risk of neurodegenerative diseases and this matter should be further studied to establish this association [58].

### 3.8. FABP-8

FABP-8 is called myelin FABP [15]. Its chromosomal location has been established to be 8q21.3–q22.1 [16]. It is associated with the peripheral nervous system, is engaged in lipid homeostasis maintenance in glial cells and constitutes a component of myelin [59]. FABP-8 has been proven to take part in the remyelination process after nerve damage and antibodies against this molecule play a role in the progression of inflammatory neuropathies [59].

FABP-8 has been investigated in psoriatic patients for the first time by our team. We also analyzed it in subjects with vitiligo and lichen planus. Its serum concentration turned out to be below detection levels in all the mentioned dermatoses. As for now, these results are currently being described to be published in original papers in the future. Our preliminary results potentially discourage the use of FABP-8 as a serum comorbidity marker in the mentioned dermatoses, but perhaps it could be investigated in larger cohorts.

### 3.9. FABP-9

FABP-9 is called testis FABP. It has been localized within the tissue of testes and salivary and mammary glands, with an encoding gene on 8q21.13 [16]. It is suggested that FABP-9 has antioxidative properties which exert a protective influence on the sperm [16].

To the best of our knowledge, FABP-9 has never been studied in psoriasis; therefore, more studies are needed to establish the potential role of this protein in psoriatics. FABP-9’s antioxidative influence has been shown to be beneficial in relation to human sperm; however, it would be interesting to investigate it in this specific group of dermatological patients. Psoriasis is known to be associated with oxidative stress [60]; hence, FABP-9 could potentially play a protective role in such patients.

### 3.10. FABP-12

FABP-12 is present in human retinoblastoma cell lines, rodent retina, and testes [61]. As this protein was relatively recently discovered, compared to other FABPs, there is no sufficient information on its role [16].

To the best of our knowledge, FABP-12 has never been studied in psoriasis, and considering in humans it has been so far found only under pathological conditions, namely retinoblastoma, its role in different disorders is uncertain.

Table 1 presents the summary of all FABPs isoforms in psoriatic patients.

**Figure 2:**
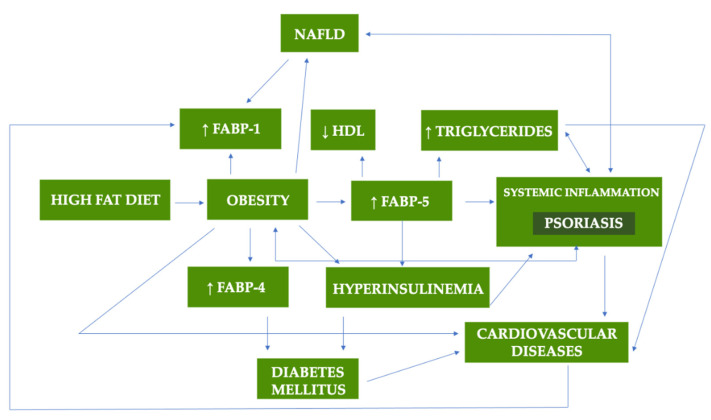
The potential role of FABPs in metabolic disorders and psoriasis [20,21,62,63,64].

Figure 2 presents the summary of potential role of FABPs in metabolic disorders and psoriasis.

## 4. Fatty-Acid-Binding Proteins as Potential Therapeutic Targets

Considering the role of FABPs in different disorders, there have been attempts to introduce drugs targeting these proteins [65]. They were investigated as a potential therapy in obesity, diabetes mellitus, atherosclerosis, or tumor metastases [65]. The following substances were taken into consideration: niacin, aryl-quinoline, urea, quinoxaline derivatives, and several more [65]. As for drugs commonly used in daily medical practice, there are reports on the lowering effect of statins, angiotensin II receptor blockers, or sitagliptin, and the elevating influence of pioglitazone on FABP-4 concentration [36]. There was also a study on the impact of anti-TNFα and anti-IL-6 agents on FABP-3, but it was performed on the group with rheumatoid arthritis [66]. After administration of adalimumab and tocilizumab, as well as methotrexate to some subjects, FABP-3 levels were higher compared to the control group [66]. The authors concluded that heart damage progresses in patients with rheumatoid arthritis and this matter should be investigated on a larger sample size [66].

We are aware of only two studies involving FABP inhibition in psoriatics. In the first study, inhibition of FABP-5 in keratinocytes in psoriatic skin led to their reduced differentiation [42]. In the second study, a topically applied molecule called VX-509 led to decreased expression of FABP-5 in the epidermis and reduced inflammation in the psoriatic skin of mice [67]. Apparently, VX-509 blocks transmission via IL-22 and activation of Stat3, which is associated with FABP-5 promoters [67].

## 5. Materials and Methods

We performed a discursive review on the possible role of FABPs in psoriasis. We searched the PubMed database between 15 and 20 May 2022 using the following MeSH: ‘psoriasis’ AND ‘fatty acid-binding protein’ OR ‘liver fatty acid-binding protein’ OR ‘intestinal fatty acid-binding protein’ OR ‘muscle heart fatty acid-binding protein’ OR ‘liver fatty acid-binding protein’ OR ‘adipocyte fatty acid-binding protein’ OR ‘epidermal fatty acid-binding protein’ OR ‘psoriasis associated fatty acid-binding protein’ OR ‘ileal fatty acid-binding protein’ OR ‘brain fatty acid-binding protein’ OR ‘myelin fatty acid-binding protein’ OR ‘FABP’ with consecutive numbers. Papers in the following languages were considered: English, Polish, German, and French, without date limitations. The whole paper was read if the abstract indicated the relevance of the content. In total, 56.7% of references come from the last 5 years.

## 6. Conclusions

FABPs have gained much attention in psoriatic patients and almost all of them have already been investigated in such patients. FABPs have different organ and tissue expression, hence various functions, and may be markers of different disorders. Considering a few of them tend to be beyond normal limits in psoriasis, it confirms the current perception of psoriasis as a multiorgan disorder associated with plenty of comorbidities. Some FABPs may be also further investigated as biomarkers of psoriasis organ complications. FABP-1 and FABP-5 may become potential markers of metabolic complications and inflammation in psoriasis. FABP-7 could perhaps be further investigated as an indicator of the neurodegenerative process in psoriatic patients. Studies on FABP-4 and FABP-5 brought inconsistent results; hence, it could be advisable to perform new research on larger cohorts to establish their exact role in psoriasis. The role of FABP-6, FABP-9, and FABP-12 still remains to be elucidated. The measurement of FABPs in psoriatic patients may help in the early prediction of comorbidities and their treatment. Moreover, they could perhaps become future therapeutic targets. New studies focusing on the changes in FABP concentrations due to administered therapy are necessary to shed some light on new treatment possibilities.

## Figures and Tables

**Table 1:** Summary of FABP isoforms in relation to psoriasis and their serum concentrations.

FABP Isoform	Name and Origin	Investigated in Serum in Psoriatics So Far	Key Observation
FABP 1 (L-FABP)	liver	Yes [21]	↑
FABP 2 (I-FABP)	intestinal	Yes [30]	↑
FABP 3 (H-FABP)	muscle and heart	Yes [20,34]	~
FABP 4 (A-FABP)	adipocyte	Yes [20,34]	↑ [20] /~ [34]
FABP 5 (E-FABP)	epidermal (psoriasis-associated)	Yes [43,44]	↑/~
FABP 6 (IL-FABP)	ileal	No	-
FABP 7 (B-FABP)	brain	Yes [58]	↑
FABP 8 (M-FABP)	myelin (peripheral myelin protein 2)	Yes [Author’s own investigation, yet to be published]	below detection level
FABP 9 (T-FABP)	testis	No	-
FABP 12	presence shown in human retinoblastoma cell lines, rodent retina, and testis	No	-

↑ means elevated serum concentration, ↓ means decreased serum concentration, ~ means similar serum concentration compared to subjects without dermatoses.
